# Reduction of corneal epithelial thickness during medical treatment for myopic regression following FS-LASIK

**DOI:** 10.1186/s12886-020-01570-2

**Published:** 2020-07-18

**Authors:** Ik-Hee Ryu, Wook Kyum Kim, Myoung Sik Nam, Jin Kook Kim, Sun Woong Kim

**Affiliations:** 1 B & VIIT Eye Center, Seoul, South Korea; 2 Department of Ophthalmology, Yonsei University Wonju College of Medicine, 20, Ilsan-ro, Wonju, Gangwon-do 26426 South Korea

**Keywords:** Corneal thickness, Corneal epithelial thickness, FS LASIK, Myopic regression

## Abstract

**Background:**

To evaluate corneal epithelial thickness change during medical treatment for eyes with myopic regression after femtosecond laser-assisted in situ keratomileusis (FS-LASIK).

**Methods:**

This study included 84 eyes of 54 subjects diagnosed with myopic regression after FS-LASIK and treated using topical steroid and anti-glaucoma drugs. Corneal epithelial thickness was measured using Fourier-domain optical coherence tomography before and after treatment. Subjects were divided into three groups based on their corneal epithelial thickness at the time of myopic regression and regression analyses were used to investigate the association between corneal epithelial thickness, visual acuity, and refraction. Logistic regression and receiver operating characteristic (ROC) curve analysis was performed to determine whether corneal epithelial thickness could predict the success of treatment, improvements of ≥ two lines in uncorrected distance visual acuity and ≥ 0.5 diopter in refraction or K.

**Results:**

Corneal epithelial thickness decreased with greater change in the center as myopic regression subsided during medical treatment. Subgroup with the thickest epithelium (≥ 62 μm) showed a higher success rate and greater changes in refraction and vision. Reduced magnitude of corneal epithelial thickness showed significant correlations with changes of K and refractive error (all *P* < 0.001). Corneal epithelial thickness was a significant factor for the success of treatment and ROC curve showed that corneal epithelial thickness > 60.50 μm had 81.5% sensitivity and 84.2% specificity for the success of medical treatment.

**Conclusion:**

Corneal epithelial thickness decreases proportionally with the magnitude of improvement of myopic regression during treatment with steroid and anti-glaucoma drugs in post-LASIK eyes with myopic regression.

## Background

Laser-assisted in situ keratomileusis (LASIK) is widely accepted as a reproducible and effective surgical procedure for correcting myopia. Surgical equipment and outcomes for LASIK have been much improved over the past few decades; however, myopic regression is still a matter of concern [1, 2]. Previous studies have suggested a forward shift of the cornea, resulting from the compromised biomechanical rigidity, as one possible explanation for myopic regression after LASIK [2, 3]; however, the underlying mechanism of refractive regression is as yet unclear. Studies have reported a relationship between the elevation of intraocular pressure (IOP) and corneal protrusion, and demonstrated that topical anti-glaucoma eye drops were effective in correcting approximately 0.5 diopter (D) of refractive regression. This was presumably a result of lowered IOP leading to the backward movement of the cornea and the flattening of its curvature [4–7]. On the other hand, steroid eye drops have long been used to decrease myopic regression after photorefractive keratectomy (PRK), as myopic regression has been known to be related to wound healing [8, 9].

The recent availability of corneal epithelial imaging by Fourier domain optical coherence tomography (FD-OCT) provides a practical tool for in vivo epithelial mapping, which demonstrates good repeatability in both normal and post-LASIK eyes [10]. It allows for the non-invasive measurement of corneal epithelial thickness in the clinical routine with adequate speed and resolution. Previous studies have reported an increase in corneal epithelial thickness following laser ablative myopic surgeries, such as LASIK [11], PRK [12], and Small incision lenticule extraction (SMILE) [13]. Studies have suggested that epithelial thickening was associated with myopic regression after LASIK as well as PRK, although the wound healing process would be quite different between both procedures [14–16]. Changes in stromal thickness have also been suggested as being responsible for postoperative refractive regression [17]. To the best of our knowledge, no studies have documented corneal epithelial thickness changes during medical treatment of myopic regression. Moreover, there are only a few studies demonstrating the widely accepted association between epithelial thickening and refractive myopic regression [18–20]. This study, therefore, aimed to determine the relationship between refractive regression and changes in epithelial thickness using FD-OCT during medical treatment of myopic regression.

## Methods

### Patients

This retrospective observational study included 84 eyes of 54 subjects that had been treated with steroid and anti-glaucoma eye drops for myopic regression, occurring after LASIK, between November 2017 and May 2019. The original surgery included femtosecond-LASIK (FS-LASIK) for the correction of myopia at the B & VIIT Eye Center (Seoul, Korea) from 2004 to 2016. Untreated post-LASIK subjects whose epithelial thickness have been followed-up for 12 weeks were presented as a control group to clarify that corneal epithelial thickness change was caused by drops in treatment group. The surgical units used for each case were variable. All patients had reached at least 20/20 uncorrected visual acuity (UDVA) 1 month after surgery and maintained stable visual acuity and refraction for at least 6 months. Preoperative data and patient characteristics are summarized in Table 1. All subjects were evaluated with manifested refraction, auto refract-keratometer (ARK-1, NIDEK, Tokyo, Japan), and Pentacam tomography (OCULUS Optikgeräte, Wetzlar, Germany). Keratometric values measured using ARK and posterior curvature with Pentacm were used to estimate corneal power change before and after medication. Myopic regression was defined as a myopic shift of 0.5 D or greater in manifest refraction from the third postoperative month following refractive surgery. The inclusion criteria of this study were as follows: 1) regression of myopia with spherical equivalent refraction of − 0.5 D or over for at least 3 months, 2) a corrected distance visual acuity of 20/20 or better, and 3) 3 months of medical treatment (10 to 14 weeks) for myopic regression. The exclusion criteria were as follows: 1) subjects with suspected under-correction during the original surgery, 2) a history of ocular surgery other than refractive surgery, 3) eyes that had had contact lenses worn in the past 1 month, 4) eyes with myopia caused by lenticular change and 5) eyes with postoperative complications including an iatrogenic ectasia. All subjects were prescribed with either loteprednol etabonate (Lotemax, Bausch & Lomb, Rochester, NY, USA) or flumetholone (Santen, Tokyo, Japan) with dorzolamide/timolol (Cosopt, Merck & Co., Inc., Whitehouse Station, NJ, USA). Patients were instructed to use a topical anti-glaucoma eye drop twice a day and a steroid eye drop four times a day for the first month and twice a day for the following 2 months.

**Table 1:** Patient demographic characteristics

	Post-LASIK (*n* = 84)
Age at operation (years)	25.0 ± 4.8
Preoperative SE (diopters)	−5.2 ± 2.1
Preoperative CCT (μm)	544.3 ± 25.7
Ablation depth (μm)	85.6 ± 27.1
Treatment duration for regression (weeks)	12.2 ± 1.2
Postoperative time at treatment (months)	82.7 ± 27.9

Mean ± SD values were presented

*SE* spherical equivalent, *CCT* central corneal thickness

To explore the influence of corneal epithelial thickness, all subjects were grouped into 3 groups based on their corneal epithelial thickness at the time of initiation of medical treatment, with group 1 consisting of CET ≤ 57, group 2, 58 ≤ CET ≤ 61, and group 3, CET ≥ 62. In addition, all subjects were categorized into three groups based on their response to medical treatment: 0, no response; 1, partial response; 2, good response. The good response was defined as successful medical treatment when patients showed the following criteria: improvement in visual acuity of two lines or more and reduction of myopia (refraction or K) with 0.5 D or more. The partial response was defined as having an uncorrected visual acuity of 20/40 or more with evidence of vision or refraction improvement that did not fulfill the criteria for good response. The no response group was defined as either lack of improvement of visual acuity/refraction or having a final visual acuity worse than 20/40. The study protocol was approved by the Institutional Review Board (CR 319130) and was conducted according to the tenets of the Declaration of Helsinki.

### Measurement of corneal epithelial thickness

The corneal epithelial and total thickness data were obtained at the time of initiation and termination of medical treatment using the RTVue OCT system (Optovue Inc., Fremont, CA, USA), with a corneal adaptor module set at a wavelength of 830 nm. We scanned the cornea in eight meridians by using the ‘Pachymetry + Cpwr (corneal power)’ scan (software version A6.11.0.12) over a 6-mm diameter centered at the corneal vertex. The corneal epithelial thickness map was generated using an automatic algorithm and was divided into a total of 17 sectors: a central 2-mm diameter zone, eight paracentral zones within an annulus between the 2- and 5-mm diameter rings, and eight mid-peripheral zones within an annulus between the 5- and 6-mm diameter rings. Stromal thickness was obtained by subtracting the epithelial thickness from the corneal thickness.

### Statistical analysis

Statistical analyses were performed using SPSS version 21.0 for Windows (IBM Corp, Armonk, NY, USA). Snellen visual acuity was converted to the logMAR scale for statistical analysis. Corneal epithelial thickness and other continuous variables obtained before and after medical treatment were compared using paired t-tests. Student t-tests were performed for comparison between treatment group and no treatment group. Subgroup analyses were conducted using one-way analysis of variance. Simple linear regression analyses were performed to investigate the association between corneal epithelial thickness and refraction, keratometric values, and UDVA (logMAR). A multivariable logistic regression analysis was performed to investigate factors affecting the success of medical treatment.

A receiver operating characteristic (ROC) curve analysis was conducted to evaluate the ability of corneal epithelial thickness to predict the success of treatment. In the ROC curve, the true positive rate (Sensitivity) was plotted as a function of the false positive rate (1-Specificity) for different cut-off points of corneal epithelial thickness at the time of medical treatment initiation. Statistical significance was defined as *P* < 0.05.

## Results

### Comparison of corneal epithelial thickness during medical treatment for myopic regression in post-LASIK eyes

**Fig. 1:**
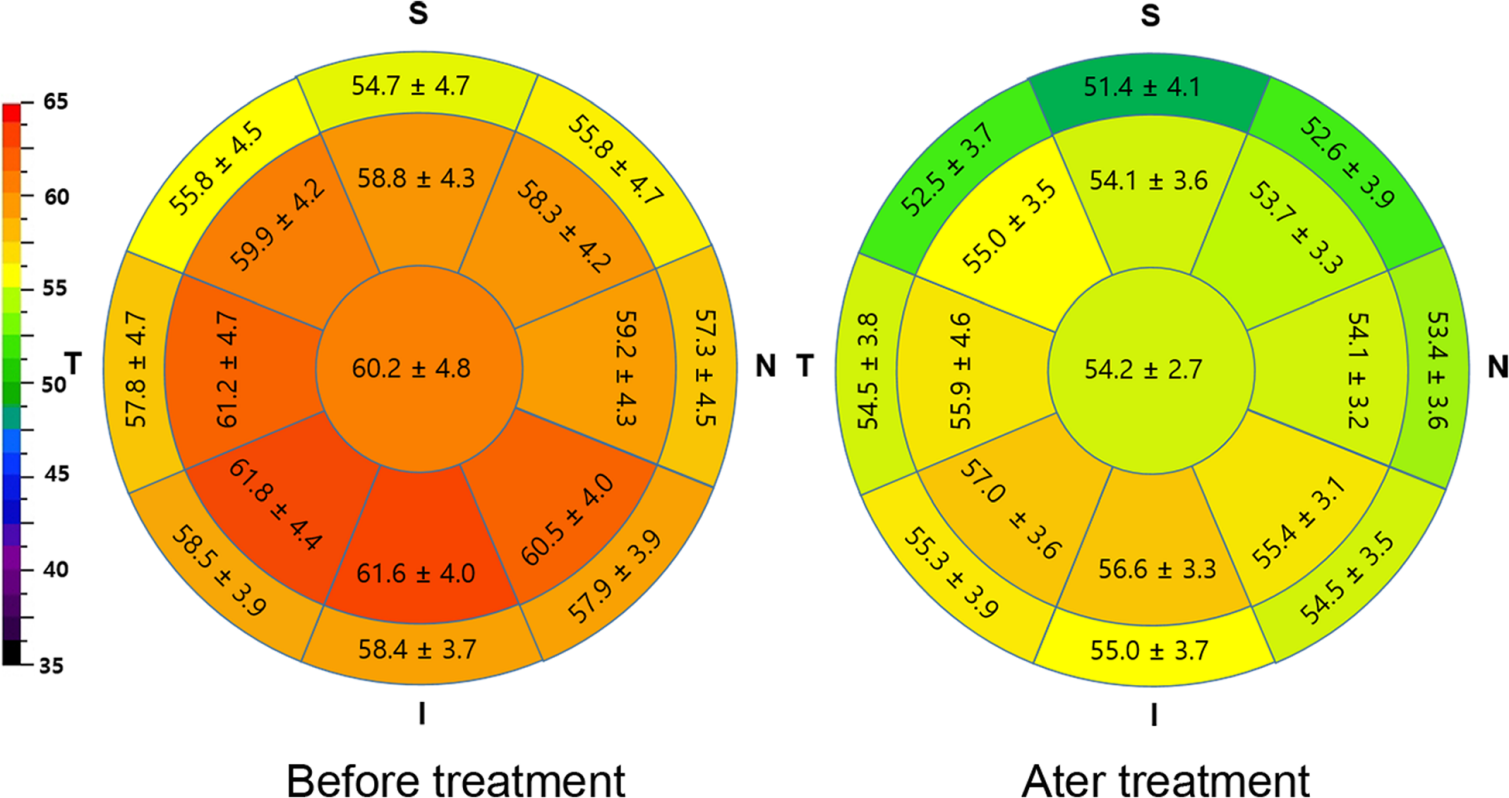
Comparison of topographic corneal epithelial thickness map before and after medical treatment for myopic regression after FS-LASIK. Myopic regressed eyes showed a thicker epithelium and decreased over 6.0 mm after medical treatment with the greatest decrease in the center (all 17 area *P* < 0.05)

The study included 84 eyes of 54 subjects undergoing medical treatment for myopic regression after FS-LASIK for myopia correction. Patient demographic data are summarized in Table 1. The average postoperative time of medical treatment initiation for myopic regression, defined as − 0.5 D or more myopia in manifest refraction, was 82.7 ± 27.9 months. Overall, there were improvements in K and UDVA at an average of 0.26 ± 0.36 D and 0.08 ± 0.13 logMAR, respectively. The corneal epithelial thickness of treatment group decreased by 5.9 ± 3.6 μm during medical treatment, whereas that of untreated eye group showed no significant changes during follow-up. Of note, central stromal thickness did not change significantly during medication (Table 2). Topographically, a greater decrease of epithelial thickness was observed in the central zone than in the paracenter, recovering to a similar topographic pattern with the usual post-LASIK epithelial thickness map after medical treatment (Fig. 1). Subgroup analysis based on the initial corneal epithelial thickness at the time of medication for myopic regression showed significant differences in changes of refraction, UDVA, and corneal power (K) between groups, while no difference in IOP change was observed. Patients with thicker corneal epithelium tended to show a greater change in corneal epithelial thickness as well as in refraction or in UDVA leading to higher success rate (Table 3). Subgroup analysis based on the response to medical treatment indicated that initial central epithelial thickness was a significant discriminating factor among various clinical parameters. Good responder group, which showed a greater reduction of epithelial thickness and greater reversals of myopic shift, had a thicker epithelium before medical treatment. Age at the operation was younger in no response group than in other groups (Table 4).

**Table 2:** Comparison of change in various ocular properties between medical treatment group and no treatment control for myopic regression in post-LASIK eyes

	Medical treatment group (*n* = 84)			No treatment control (*n* = 33)			P*
	Initial	End	*P*	Initial	End	*P*	
Sphere	−1.04 ± 0.40	−0.88 ± 0.51	0.001	− 0.20 ± 0.40	−0.34 ± 0.46	0.012	< 0.001
(D)	(−2.0, **−** 0.50)	(− 2.3, 0.50)		(− 1.0, 0)	(−1.25, 0)		
Cylinder	−0.46 ± 0.30	−0.49 ± 0.25	0.251	−0.44 ± 0.23	−0.48 ± 0.28	0.226	0.840
(D)	(−1.5, 0)	(−1.25, 0)		(−1.0, 0)	(− 1.5, 0)		
SE (D)	−1.26 ± 0.41	−1.13 ± 0.51	0.010	−0.42 ± 0.39	−0.58 ± 0.45	0.004	< 0.001
	(− 2.25, − 0.63)	(− 2.63, 0.38)		(−1.13, − 0.5)	(− 1.5, − 0.38)		
UDVA	0.25 ± 0.12	0.17 ± 0.15	< 0.001	− 0.07 ± 0.05	− 0.07 ± 0.05	0.801	< 0.001
(logMAR)	(0.05, 0.5)	(−0.1, 0.5)		(−0.1, 0.1)	(− 0.1, 0.1)		
IOP	11.3 ± 1.4	10.4 ± 1.8	< 0.001	11.2 ± 1.9	10.8 ± 1.5	0.062	0.084
(mmHg)	(9, 16)	(7, 15)		(8, 17)	(8, 15)		
CET (μm)	60.2 ± 4.8	54.2 ± 2.7	< 0.001	56.1 ± 3.5	55.5 ± 4.31	0.173	< 0.001
	(53, 75)	(49, 65)		(48, 63)	(47, 68)		
mET (μm)	60.0 ± 3.8	55.2 ± 3.1	< 0.001	58.6 ± 3.8	58.0 ± 4.0	0.218	< 0.001
	(53.9, 71.6)	(49.1, 65.6)		(50.1, 64.9)	(49., 69)		
pET (μm)	57.0 ± 3.0	53.7 ± 3.0	< 0.001	56.7 ± 3.1	56.7 ± 3.2	0.662	< 0.001
	(51.1, 64.3)	(47.4, 62.9)		(50.1. 63.3)	(49.1, 65)		
ST (μm)	412.9 ± 28.1	413.8 ± 27.8	0.06	411.0 ± 25.3	412.7 ± 26.9	0.102	0.620
	(352, 477)	(358, 477)		(350, 471)	(350, 474)		
K (D)	38.9 ± 1.8	38.5 ± 1.9	< 0.001	38.7 ± 1.5	38.6 ± 1.6	0.134	0.004
	(35.0, 41.8)	(34.5, 42.0)		(36.0, 41.5)	(35.8, 42)		
PC (mm)	6.34 ± 0.25	6.31 ± 0.25	< 0.001	6.34 ± 0.23	6.31 ± 0.22	0.006	0.954
	(5.93, 7.06)	(5.78, 6.97)		(5.81, 6.74)	(5.84, 6.74)		

Mean ± SD (min, max) values were presented; *SE* spherical equivalent, *UDVA* uncorrected distant visual acuity, *logMAR* logarithm of the minimum angle of resolution, *IOP* intraocular pressure, *CET* central corneal epithelial thickness, *mET* paracentral epithelial thickness, *pET* pericentral epithelial thickness, *ST* stromal thickness at center, *K* Corneal power measured using ARK, *D* diopters, *PC* posterior corneal radius of curvature measured using Pentacam. *P*: comparison between initial measurement and end measurement using paired t-test. P*: comparison of differences between treatment group and no treatment using independent t-test

**Table 3:** Comparison of characteristics between subgroups formed based on corneal epithelial thickness at the initiation of medical treatment for myopic regression after LASIK

	Group 1 CET ≤ 57 (*n* = 26)	Group 2 58 ≤ CET ≤ 61 (*n* = 32)	Group 3 CET ≥ 62 (*n* = 26)	*P*
Preop SE (D)	−4.4 ± 2.0	−4.9 ± 1.8	−6.5 ± 2.0	0.001
Age at operation	23.8 ± 5.3	24.3 ± 4.6	26.5 ± 4.4	0.070
CCTini (μm)	477.0 ± 26.6	474.2 ± 24.8	469.4 ± 27.3	0.546
CETini (μm)	55.6 ± 1.3	59.2 ± 1.1	65.9 ± 4.0	< 0.001
Good response (%)	1 (3.8)	6 (18.8)	20 (76.9)	< 0.001
No response (%)	15 (57.7)	13 (40.6)	1 (3.8)	< 0.001
SEini (D)	−1.18 ± 0.38	− 1.22 ± 0.39	−1.40 ± 0.45	0.126
dCET (μm)	−3.1 ± 2.0	−5.4 ± 2.6	−9.4 ± 3.3	< 0.001
	(−6, 1)	(− 11, 1)	(− 17, − 5)	
dSE (D)	−0.08 ± 0.35	0.04 ± 0.34	0.48 ± 0.55	< 0.001
	(− 1.25, 0.50)	(− 0.75, 0.75)	(− 0.50, 1.88)	
dUDVA	− 0.00 ± 0.11	− 0.04 ± 0.09	− 0.18 ± 0.11	0.001
	(−0.25, 0.35)	(−0.25, 0.1)	(− 0.4, − 0.05)	
dK	−0.09 ± 0.25	−0.16 ± 0.26	−0.57 ± 0.37	0.001
	(−0.75, 0.5)	(− 0.5, 0.25)	(− 1.25, 0.25)	

Mean ± SD (min, max) values were presented; *CET* central corneal epithelial thickness, *SE* spherical equivalent, *ini* initial, before treatment, *d* delta (measurement at the end – initial measurement, *UCVA* uncorrected visual acuity, *K* Corneal power measured using ARK, *D* diopters. *P*: comparison using one-way ANOVA

**Table 4:** Comparison of characteristics between subgroups formed based on responsiveness to medical treatment for myopic regression after LASIK

	No response (*n* = 29)	Partial response (*n* = 28)	Good response (*n* = 27)	*P*
Age at operation	22.8 ± 2.4	26.3 ± 6.2	25.9 ± 4.5	0.008
CETini (μm)	57.0 ± 2.2	58.9 ± 2.8	64.9 ± 4.9	< 0.001
SEini (D)	− 1.35 ± 0.39	−1.02 ± 0.30	−1.42 ± 0.43	< 0.001
Kini (D)	39.3 ± 1.6	38.8 ± 1.9	38.5 ± 1.8	0.219
dCET (μm)	−3.4 ± 2.3	−5.6 ± 2.6	−9.0 ± 3.6	< 0.001
dmET (μm)	−3.7 ± 2.0	−4.7 ± 2.5	−6.4 ± 3.2	0.001
dSE (D)	−0.19 ± 0.32	−0.02 ± 0.23	0.64 ± 0.38	< 0.001
dUDVA	0.04 ± 0.09	−0.07 ± 0.08	−0.21 ± 0.08	< 0.001
dK	0.01 ± 0.25	− 0.16 ± 0.20	−0.65 ± 0.25	< 0.001

Mean ± SD values were presented; *SE* spherical equivalent, *CET* central corneal epithelial thickness, *mET* paracentral epithelial thickness, *UDVA* uncorrected distant visual acuity, *K* Corneal power measured using ARK, *D* diopters, *d* delta (measurement at the end – initial measurement. *P*: comparison using one-way ANOVA

### Association between corneal epithelial thickness and success of medical treatment

**Fig. 2:**
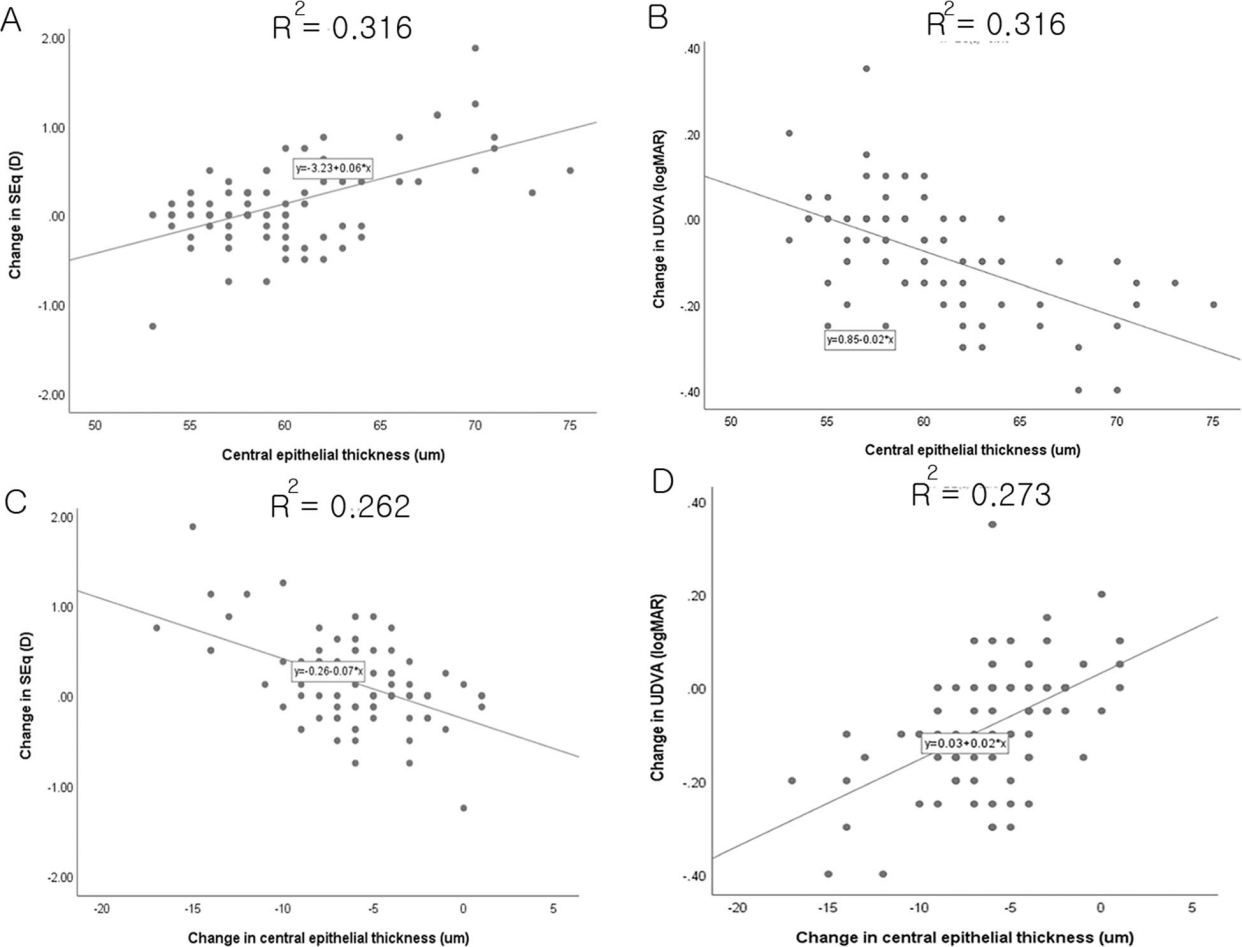
Linear correlations between central epithelial thickness of at the time of diagnosis of myopic regression after FS-LASIK and (**a**) changes in spherical equivalent (SEq) refraction and (**b**) uncorrected distant visual acuity (UDVA) during medical treatment. Linear associations were observed in (**c**) changes of corneal epithelial thickness with SEq and (D) UDVA during medical treatment

**Fig. 3:**
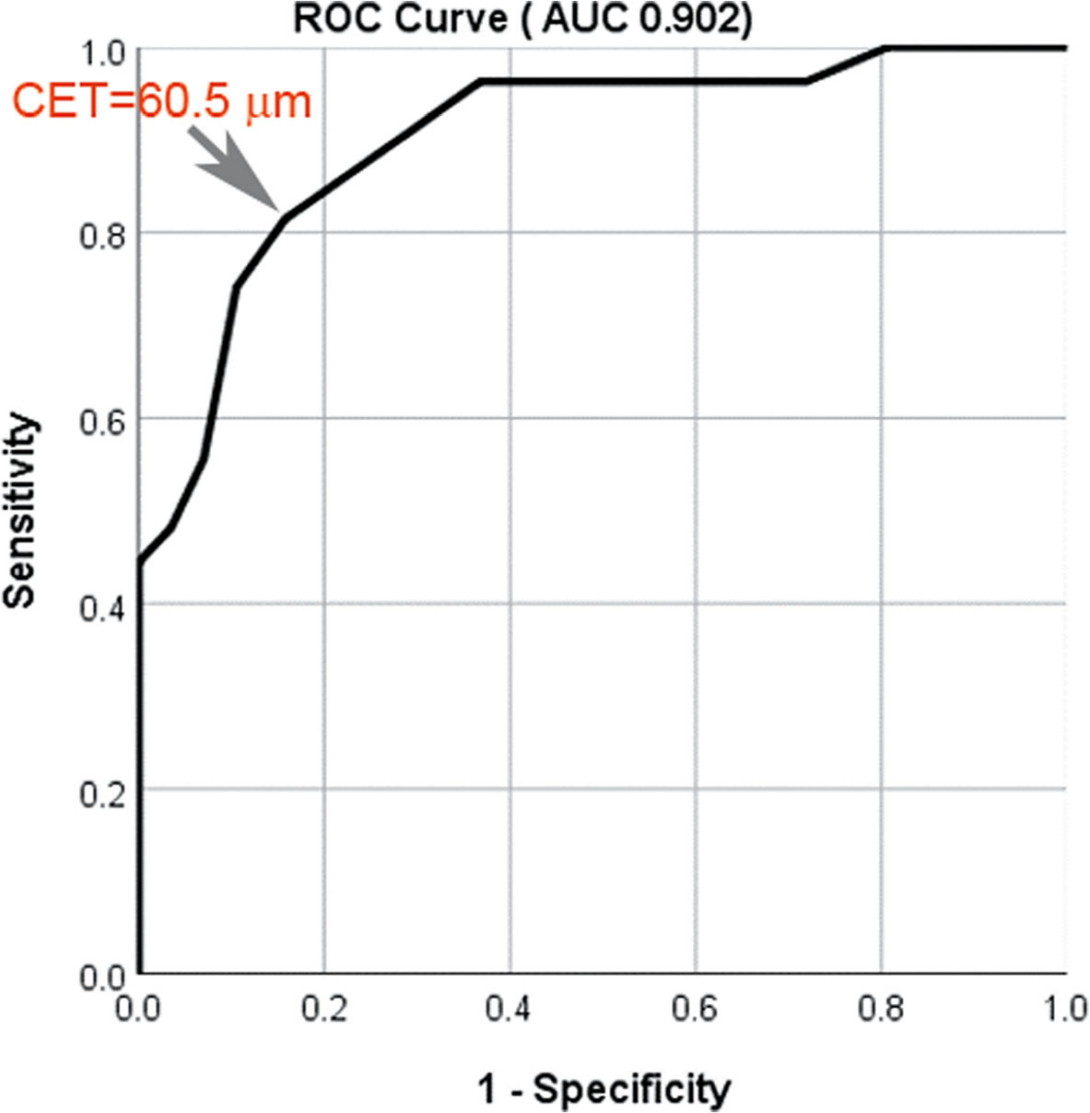
Receiver operating characteristic (ROC) curve generated from epithelial thickness to predict the success of medical treatment for myopic regression after LASIK. The area under the curve (AUC) was 0.902. Each point on the ROC curve represents a sensitivity/specificity pair corresponding to a particular threshold of corneal epithelial thickness at which an improvement in visual acuity of two lines or more with a change of 0.5 diopter or more can be expected, after medical treatment for myopic regression in post-LASIK eyes. Central epithelial thickness (CET) of 60.50 μm, with 81.5% sensitivity and 84.2% specificity, showed the highest Youden index

Simple and multiple linear regression analysis showed that corneal epithelial thickness at the beginning of medical treatment showed a significant association with the improvement of UDVA and reversal of myopic regression. Furthermore, the changes in corneal epithelial thickness during medical treatment showed a statistically significant linear association with the changes in refractive error (spherical equivalent) and UDVA (Fig. 2). Although there was a significant decrease in IOP after medical treatment, no correlation between change in IOP and changes in corneal epithelial thickness or refraction was detected. Since the degree of medical response was significantly associated with change in corneal epithelial thickness and initial epithelial thickness, we hypothesized that subjects with greater corneal epithelial thickness may be more responsive to medical treatment. To test this hypothesis, we first performed logistic regression to explore factors affecting the success of medical treatment and then evaluated the usefulness of corneal epithelial thickness as an indicator to begin medical treatment. The logistic regression analysis identified the central corneal epithelial thickness as a significant factor for good response (OR = 2.045, *P* = 0.005) after adjusting for age, preoperative SE, SE before treatment, CCT, UDVA, and IOP (Table 5). The *ROC curve* was created to predict the success of medical treatment defined as an improvement of ≥ two lines in UDVA and ≥ 0.5 diopter in refraction by plotting the true positive rate (sensitivity) against the false positive rate (1-specificity) at various levels of corneal epithelial thickness at the time of treatment initiation. As shown in Fig. 3, the area under the curve (AUC) was 0.902 and central epithelial thickness of 60.50 μm represented the highest Youden index, with 81.5% sensitivity and 84.2% specificity.

**Table 5:** Logistic regression analysis to predict success of medical treatment

	Crude		Model			
	*P*-value	OR	B	*P*-value	OR	95% CI for OR
Age at op	0.913	1.006	−0.074	0.544	0.929	0.731–1.180
CCTini	0.898	0.999	0.010	0.596	1.010	0.974–1.047
CETini	< 0.001	1.594	0.715	0.005	2.045	1.246–3.355
Preop SE	0.234	0.846	0.347	0.265	1.415	0.769–2.602
SEini	0.117	0.364	−2.110	0.207	0.121	0.005–3.202
UDVAini	0.717	2.370	−7.934	0.161	0	0–23.573
IOPini	0.687	0.919	0.287	0.342	1.333	0.737–2.413

*OR* odd ratio, *CET* central corneal epithelial thickness, *CCT* central corneal thickness, *SE* spherical equivalent, *ini* initial, before treatment, *UDVA* uncorrected distant visual acuity (logMAR)

## Discussion

It is widely known that the corneal epithelium, which is highly reactive to irregularities and changes in the underlying stroma, undergoes remodeling in response to corneal refractive surgery. Studies suggest that despite preservation of corneal epithelium and Bowman’s layer, epithelial remodeling occurs after LASIK in a similar manner to that observed after PRK [11, 21]. It has been suggested that epithelial hyperplasia may contribute to myopic regression after myopic laser ablation; however, its association with refractive change is as yet unclear. Erie [20] showed that myopic regression after PRK was significantly associated with an increase in corneal epithelial thickness, whereas Ivarsen et al. [17] did not find any correlation between changes in corneal epithelial thickness and changes in refraction after PRK or LASIK. Studies have failed to demonstrate a correlation between the change in corneal epithelial thickness and refraction, presumably due to short-term follow-ups, complex predisposing factors, and a lack of reliable measurements across the longitudinal follow-ups. Notably, a recent study demonstrated that myopic shift observed at postoperative 1 year showed a significant correlation with increase of corneal epithelial thickness after PRK [18].

Other than epithelial hyperplasia, forward movement of the cornea, accumulation of new stromal collagen, or lens-induced regression has been suggested to play a role in myopic regression [1, 2], however, the mechanism for myopic regression after LASIK still remains to be elucidated. Of these suggested mechanisms, we aimed to investigate the association between corneal epithelial thickness and myopic regression. The recent introduction of corneal epithelial thickness mapping in FD-OCT prompted us to study epithelial remodeling after laser refractive surgery and we monitored changes in corneal epithelial thickness during medical treatments for clinically diagnosed myopic regression. As a result, we have demonstrated a significant association between corneal epithelial thickness and refraction.

Previous researches described a negative meniscus-like lenticular pattern, with more thickening at the paracenter than at the center after LASIK due to aspheric ablation profiles [11, 21, 22], but myopic regressed eyes showed more epithelial thickening at the center than paracenter as shown in our data. If we compared topographic epithelial thickness pattern between no response and good response group, central hyperplasia pattern was prominent in good response group, while a negative meniscus-like lenticular pattern was observed in no response group (supplemental figure). Of note, epithelial thickness decreased at the center by the greatest amount followed by the paracenter after medical treatment, and this topographic thickness change being accompanied by refractive changes was in agreement with Barraquer’s law of thicknesses which indicates that if there is relative tissue addition in the center of the cornea, and conversely if there is relative tissue removal from the center of the cornea there will be a hyperopic refractive shift [23]. As a result, overall topographic epithelial thickness pattern over 6.0 mm after medical treatment was recovered to a negative meniscus-like lenticular pattern, similar to the previously published postoperative map [11, 21, 22].

Our study raised some issues related to myopic regression and epithelial remodeling. First, this study suggested that further increase in central epithelial thickness may occur years after LASIK in some eyes. Our data showed that central epithelial thickness of regressed eyes were significantly thicker than untreated eyes where the refraction has been stable long-term (Table 2). Of note, our data demonstrated that eyes with thicker epithelium have been treated for greater myopia. Second, epithelial thickening could, at least, partly be reversed by medical treatment. It was a surprise to observe active epithelial remodeling, years after surgery that was responsive to medical treatment.

To the best of our knowledge, this study is the first to report that decrease in corneal epithelial thickness occurs during medical treatment and that there is an association with improvement in visual acuity and refraction. This study, therefore, reinforces the role of epithelial thickening in the development of myopic regression following myopic LASIK.

There are limitations in this study. First, we were unable to clarify which medication showed the observed effect due to absence of solitary anti-glaucoma drop treatment group. We hypothesized that topical steroid may have epithelium modulating effect and added anti-glaucoma drops to prevent the possible IOP rise during steroid treatment, but did not include an anti-glaucoma drop treated group in this study. Previous studies have reported the effects of steroid and anti-glaucoma eye drops in reversing myopic regression after PRK and LASIK, respectively [5–9]. A previous study concluded that topical steroids did not seem to have a beneficial role in routine postoperative treatment after LASIK [24]; however, no studies have evaluated the effect of steroids on myopic regression after LASIK. Studies have shown that steroid eye drops delay proliferation and attenuate migration of corneal epithelial cells [25, 26]. It is conceivable that steroid retard an epithelial proliferation and migration for subjects having a greater epithelial hyperplastic response and decrease (normalize) epithelial thickness; however, the exact mechanism needs to be clarified in further studies. Topographically, a greater decrease of epithelial thickness was observed in the center than in the paracenter. This result may be supporting our hypothesis because central hyperplastic area may demand more proliferation and migration to maintain thickness. With regard to possible effects of anti-glaucoma drops on myopic regression, we could not observe the suggested finding by previous publications, the backward movement of protruded cornea by lowering the IOP [3, 5]. In our analysis, however, flattening of the cornea has only been shown with the anterior curvature, not posterior curvature (Table 2). Furthermore, we could not observe the relationship between the magnitude of decrease of IOP and refraction change. Second, this study did not contain important data such as duration of the period over which reversal was maintained or recurrence rate after discontinuation of medication. In our experience, about 80% patients showed a stable refraction for 3 months after termination of eyedrops, but no long-term follow-up data is available. In the context of usefulness as a treatment option for myopic regression, this study lacks the data to address critical questions. A prospective study is necessary to optimize treatment strategy and to investigate how to maintain stable refraction after initial success. Third, clinical implication of our cut-off epithelial thickness may be very limited because we could not assess the real magnitude of epithelial hyperplasia due to unavailable preoperative measurement. Despite these limitations, we tested the feasibility of corneal epithelial thickness as an indicator for the initiation of medical treatment for myopic regression. Our data showed that with central corneal epithelial thickness > 60.50 μm, there was an expected improvement in visual acuity of more than two lines and 0.5 D or greater change in refraction with about 80% sensitivity and specificity. We believe that measurement of baseline epithelial thickness and longitudinal follow up could provide a better strategy for myopic regression. Based on this finding, we suggest that medical treatment may be beneficial for patients with thicker corneal epithelium and myopic regression.

## Conclusion

The major clinical implication of this study was to demonstrate a significant association between change in corneal epithelial thickness and refraction. Our data suggested that decrease in corneal epithelial thickness occurred concurrently with flattening of corneal curvature, leading to an improvement in refraction and visual acuity. Furthermore, this study suggested that corneal epithelial thickness plays a role in deciding whether patients would respond to medical treatment. We believe that routine measurement and monitoring of corneal epithelial thickness could be useful in subjects seeking laser refractive surgery to understand the pathogenesis of myopic regression as well as to optimize treatment strategy.

Interestingly, this study demonstrated that corneal epithelial thickness decreased as myopic regression subsided, and the change in corneal epithelial thickness was significantly associated with refractive change.

## Supplementary information

**Additional file 1: Supplemental Figure.** Comparison of topographic corneal epithelial thickness maps of no response myopic regressed eyes and good response myopic regressed eyes.

## Data Availability

The datasets used and/or analyzed during the current study are available upon reasonable request. Confidential patient data will not be shared.

## Abbreviations

FS-LASIK: Femtosecond laser-assisted in situ keratomileusis
ROC: Receiver operating characteristic
UDVA: Uncorrected distance visual acuity
IOP: Intraocular pressure
PRK: Photorefractive keratectomy
FD-OCT: Fourier domain optical coherence tomography
SMILE: Small incision lenticule extraction
