# Supplementary figures and images for: Reduction of corneal epithelial thickness during medical treatment for myopic regression following FS-LASIK
Source: BMC Ophthalmol. 2020 Jul 18;20:296. doi: 10.1186/s12886-020-01570-2 (PMC7368729; doi:10.1186/s12886-020-01570-2)

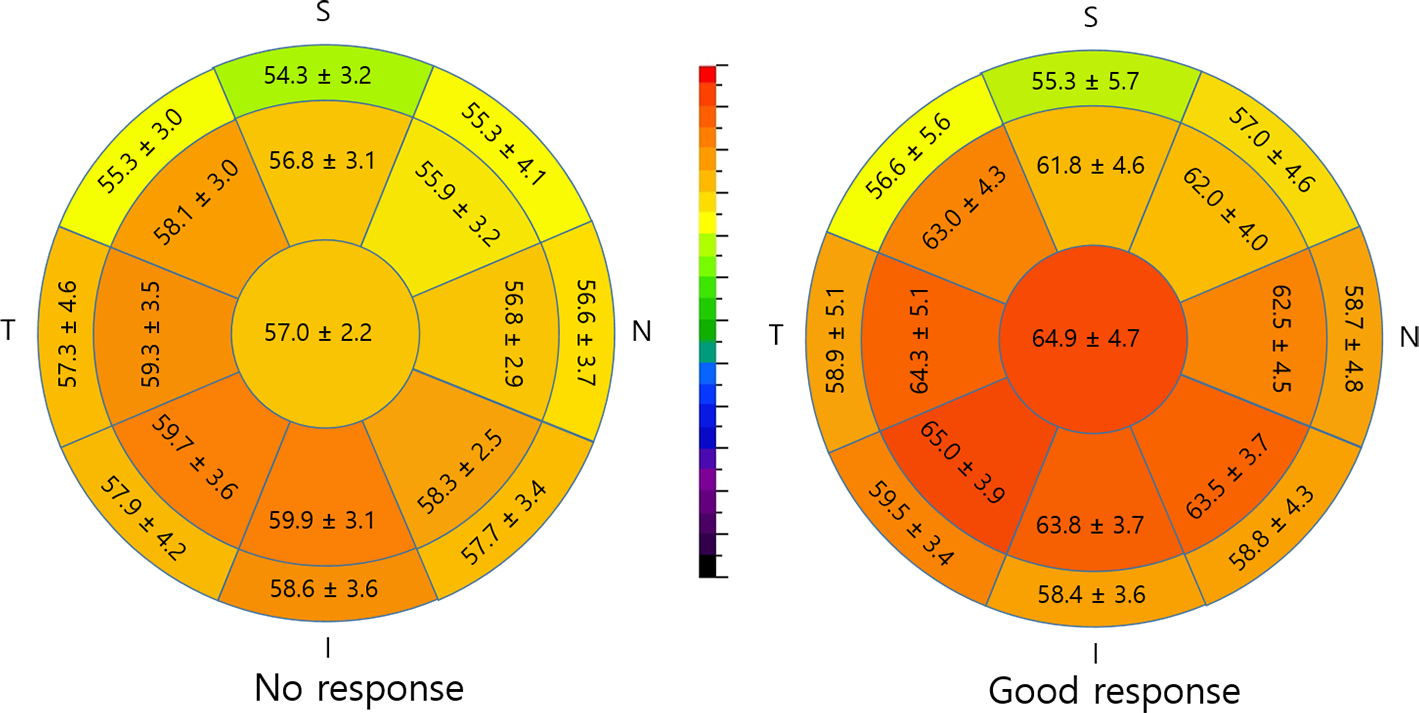

Supplement: Supplementary file 1 — Additional file 1: Supplemental Figure. Comparison of topographic corneal epithelial thickness maps of no response myopic regressed eyes and good response myopic regressed eyes. [file 12886_2020_1570_MOESM1_ESM.tif]
